# A Clinicopathological Profile of Prostate Cancer in Trinidad and Tobago

**DOI:** 10.1155/2016/2075021

**Published:** 2016-07-17

**Authors:** Ian Hosein, Rajendra Sukhraj, Lester Goetz, Nastassia Rambarran, Satyendra Persaud

**Affiliations:** ^1^Department of Urology, San Fernando General Hospital, San Fernando, Trinidad, Trinidad and Tobago; ^2^Division of Surgical Sciences, University of the West Indies, St. Augustine, Trinidad, Trinidad and Tobago; ^3^The 4H Hospital, Bridgetown, Barbados

## Abstract

*Aim.* To conduct a clinicopathological review of all prostate biopsies performed in a tertiary referral centre in Trinidad and Tobago over a period of 30 months.* Methods.* The records of all patients who had prostate biopsies from January 2012 to July 2014 were reviewed. Clinical and pathologic data were compiled and subsequently analysed using SPSS version 20.* Results.* From January 2012 to July 2014, 617 transrectal ultrasound guided prostate biopsies were performed. Pathological data were found for 546 patients of whom 283 (51.8%) were confirmed carcinoma of the prostate. Moderately differentiated tumors (Gleason 7) were the most common group. Using the D'Amico risk classification, most cases were found to be high risk (63.1%). Afro-Trinidadians comprised 72.1% of the patients with prostate cancer. Afro-Trinidadians were also more likely to have high risk and high grade disease as well as high PSA values.* Conclusion.* This study demonstrates that over half of our biopsies are eventually positive for cancer and most cases were high risk. Afro-Trinidadians comprised a disproportionate number of those diagnosed with prostate cancer and had a greater risk of high risk disease.

## 1. Introduction

The incidence and mortality of prostate cancer in the Caribbean are among the highest in the world [1]. Trinidad is an ethnically diverse country with the population consisting primarily of Indo-Trinidadians (40%) and Afro-Trinidadians (37%) [2]. Prostate cancer is the leading cause of cancer-related mortality among men, accounting for 38% of these deaths and is therefore a significant public health concern [3]. Yet much remains to be documented about the disease locally.

The Department of Urology at San Fernando General Hospital (UDSFGH) is a tertiary referral centre which serves a population of 650,000 but routinely receives patients from throughout Trinidad and Tobago. It is a Societe Internationale d'Urologie (SIU) approved training facility. The department is affiliated with the University of the West Indies and there are currently 9 residents undergoing postgraduate training in urology. At present, the department is staffed by 20 doctors including 4 consultants.

Previous work at our institution noted that most men with prostate cancer were classified as high risk and that Afro-Trinidadians were at highest risk of developing prostate cancer [4]. This is also in keeping with data generated by our national cancer registry [3]. Our study builds on this research as it represents the first analysis in which the cohort was uniformly assessed using a 12-core ultrasound guided biopsy technique which meets international standards of care.

## 2. Patients and Methods

All patients who underwent transrectal ultrasound (TRUS) guided prostate biopsy between January 2012 and June 2014 were retrospectively identified and their details were entered into a database. Clinicopathological data were collected including age, address, race, serum PSA, pathological diagnosis, and clinical stage. In the case of prostate cancer, the Gleason score and histological subtype were also recorded.

All biopsies were performed by trained urology residents and were conducted under ultrasound guidance using an 8 MHz transrectal probe (B-K Medical Systems). A 12-core double sextant pattern was followed utilizing a Bard biopsy gun and 18 G core biopsy needle. Pathological analyses were conducted by specialist pathologists primarily within our institution using routine Hematoxylin and Eosin (H and E) stains. However, we did include analyses done by certified pathologists outside the institution if the patient had opted for this route.

Data were collected and compiled in MS Excel. PSA levels were categorized instead of treated as continuous variables because various laboratories used different cutoff levels for reporting exact PSA numbers. Tests of association between categorical variables were performed using Pearson's chi-square test and Fisher's exact test with* p* value of <0.05 being considered as statistically significant. Statistical analysis was performed using SPSS version 20.

## 3. Results

### 3.1. Patient Demographics

During the 30-month period, 617 biopsies were performed. Pathological data were found for 546 patients and data analysis was therefore limited to these cases. Age data were available for all but 2 patients and, overall, the mean age of the population was 68.6 years (SD = 8.6, range 44–90). Afro-Trinidadians comprised 68.3% and Indo-Trinidadians 29.7%. Among patients with prostate cancer, the mean age was 69.4 years (SD = 8.1, range 46–90); Afro-Trinidadians comprised 72.1% and Indo-Trinidadians 25% of these 283 patients.

### 3.2. Histological Analysis

**Figure 1:**
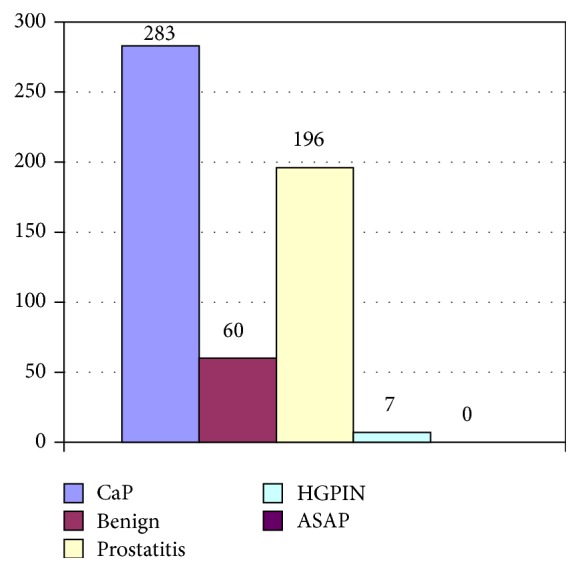
Distribution of the patients by histological diagnosis, *n* = 546.

Of the 546 patients for whom histological data could be found, 283 (51.8%) were diagnosed with prostate cancer. Isolated high grade prostatic intraepithelial neoplasia (HGPIN) was found in 7 cases (1.3%). Additionally, HGPIN was noted as a secondary histological finding in 7 patients with prostate cancer, making the overall incidence of HGPIN 2.6%. No case of atypical small acinar proliferation (ASAP) was found. There were 196 cases of prostatitis. The histological diagnoses are summarized in Figure 1. Histologically, all cancers were acinar adenocarcinoma. Moderately differentiated tumors were most common (45.6%), while well differentiated tumors accounted for only 15.2% (Table 1). Among 129 tumors with a Gleason score of 7, 77 tumors (59.7%) were assigned a score of 3 + 4, while 52 (40.3%) were assigned a score of 4 + 3.

### 3.3. PSA and Clinical Stage

**Figure 2:**
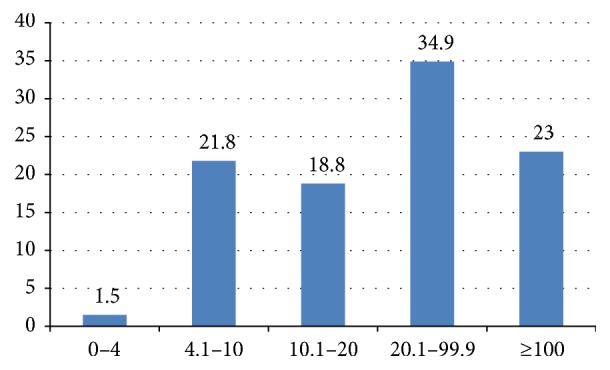
Distribution of PSA values among patients with prostate cancer, *n* = 261.

PSA data were available for 261 of the 283 patients with prostate cancer. PSA values among the patients with prostate cancer are depicted in Figure 2. 22.9% had a PSA greater than or equal to 100 ng/mL. Afro-Trinidadians were more likely than their Indo-Trinidadian counterparts to have a PSA ≥ 100 ng/mL (*p* < 0.001). Among the 261 patients for whom clinical stage was recorded, most were stage T2 (46.4%) or T1c (36.4%).

### 3.4. Risk Classification

Due to incomplete data, 36 (12.7%) patients could not be assigned a risk status, so analysis was therefore confined to 247 patients (Table 2). Using the D'Amico risk classification, most cases were found to be high risk (63.1%) followed by intermediate risk (29.6%) and low risk (7.3%). Afro-Trinidadians were noted to be at an increased risk of having high risk disease (68%) when compared to men of Indo-Trinidadian ancestry (50.8%).

### 3.5. Impact of Race

As described earlier, Afro-Trinidadians comprised the majority (72.1%) of the prostate cancer population. We found that patients of African descent were more likely to have higher PSAs compared to their Indo-Trinidadian counterparts (Table 3); 27.5% of Afro-Trinidadians had a PSA > 100 ng/mL compared to 10.6% of Indo-Trinidadians (*p* < 0.001). Afro-Trinidadians were more likely to have poorly differentiated tumors compared to Indo-Trinidadians—44% compared 25% (*p* = 0.01). Perineural invasion was more common among Afro-Trinidadians (11.3%) when compared to Indo-Trinidadians (5.6%), but this did not attain statistical significance (*p* = 0.353). As previously mentioned, Afro-Trinidadians were more likely to have D'Amico high risk tumors (Table 2).

## 4. Discussion

Malignancies accounted for 51.8% of our biopsies. In a Saudi Arabian study, the rate of malignancy on biopsy was noted to be 17.7%, while, in a review of biopsies done in Nigeria, the authors found that 28.9% were malignant [5, 6]. Similar studies in the United Kingdom and the United States have documented rates of malignancies on extended core biopsies of 33% and 44% [7, 8]. Although this illustrates that rates vary globally, the rate of malignancies in our study would be considered high by any standard. One potential explanation for this may be the paucity of organized screening in the Caribbean and as a result many patients still present late. Earlier work in Jamaica noted that between 50% and 80% of men still present with symptomatic disease [9, 10].

Further evidence of late presentation may lie in the clinical stage and PSA values noted among our patients. We noted that low stage T1c tumors accounted for only 36% of our patients compared to 76.1% in a recent US study [11]. In addition to this, another difference between our study and other contemporary series is the high proportion of patients with markedly elevated PSA values (greater than or equal to 100 ng/mL) which occurred in 22.9% of our patients. However, similar figures have been noted by researchers in Jamaica; Coard and Skeete found that 31.6% of their patients had PSAs ≥ 100 ng/mL [12]. While work in the USA has shown that the mean PSA is around 5.7 ng/mL, only 22% of our patients had PSAs in the range of 4.0 ng/mL–10 ng/mL [11]. Disparate mortality rates have been noted between Caribbean and North American men, with men from the Caribbean having lower 5-year survival. However, no difference was noted between immigrant Caribbean men and Afro-American men [13]. This is hypothesis generating as it suggests that interventions aimed at earlier detection among Caribbean men may be beneficial. At present, the Caribbean region has no screening guidelines, although recent work by Aiken and Persaud has demonstrated that most regional urologists do favour screening and are of the opinion that guidelines are necessary [14].

All of our cases were acinar adenocarcinomas. This is not surprising given the rarity of histological variants. In a Jamaican study, one case of ductal cancer was identified among 529 malignancies reviewed, while, in the United States, analysis of the Surveillance Epidemiology and End Results (SEER) Database identified 371 ductal cancers (0.08%) among 443252 patients [12, 15].

In our study, moderately (45.6%) and poorly differentiated (39.2%) tumors were most common, while well differentiated tumors comprised only 15.2%. This is comparable to the Jamaican study in which moderately differentiated tumors (37.5%) were also noted to be the most common [12]. Similar results have been noted in the Middle East [5].

Perineural invasion (PNI) was noted in 2.6% of needle biopsies in our study. Bastacky et al. in a study of 302 biopsy specimens reported that the incidence of PNI was 20%, while, in a larger more contemporary series, Loeb et al. reported 188 cases of PNI among 1256 patients, a rate of 15% [11, 16]. However, it has been previously found in a review of the literature that the rate of HGPIN on needle biopsy varied widely, from 0% to 25% with a mean of 7.7% [17]. We also noted that no case of atypical small acinar proliferation (ASAP), a premalignant lesion, was found. This was surprising as work in Jamaica by Brown and colleagues demonstrated that the rate of ASAP on needle biopsy was 3.4% [18]. In the previously mentioned paper by Epstein and Herawi, the incidence of ASAP on needle biopsy was 5% [17]. Variation in the interobserver reproducibility of the diagnosis of ASAP between pathologists has been described and may have accounted for the absence of an ASAP diagnosis and even the low incidence of HGPIN in our study population.

When it came to the impact of race on histological findings, we found that prostate cancer was almost three times as common among Afro-Trinidadian men, who comprised 72% of the prostate cancer population, compared to Indo-Trinidadians who comprised 25%. When one considers that Afro-Trinidadians make up 37.5% of the population of Trinidad and Tobago and Indo-Trinidadians 40%, the disproportionate representation of blacks among the cancer population is even more striking. This finding has been previously reported by researchers in Trinidad and Tobago and seems to mirror global patterns of increased prostate cancer risk among black men [4]. In our study, Afro-Trinidadian men were also more likely to have poorly differentiated and high risk tumors. These findings were not entirely surprising, as much has been written about the propensity of blacks towards more aggressive disease [19–22].

Previous unpublished work at our institution calculated the incidence of prostate cancer among Indo-Trinidadians as 38/100,000. Comparably, Rastogi et al. reported an age adjusted incidence rate of 4.6/100,000 among men in India [23]. These contrasting differences in incidence may be due to differences in screening practices as well as environmental influences as highlighted by the fact that persons of Indian origin residing in developed countries have significantly higher rates of prostate cancer [23].

Firstly, this study was retrospective in nature and we were unable to retrieve all of the histology reports. Our study sampled patients from only one, albeit the major, urological referral centre in the country and individual addresses were not recorded. Our ability to accurately extrapolate our results to the wider population is therefore limited. Nonetheless, experience has shown that we routinely see patients from throughout Trinidad and Tobago and this may have mitigated the limitation somewhat. Additionally, ethnicities were self-assigned which may have been a potential limitation given that Trinidad is a multiethnic society. However, we have no reason to believe that this population heterogeneity would have influenced the outcome of this study in any significant way. Much research into the genetics of prostate cancer as well as treatment and treatment related outcome remains to be conducted.

## 5. Conclusion

This study demonstrates that prostate cancer is more common among men of African descent. Afro-Trinidadian men also had higher grade and higher risk disease than Indo-Trinidadian men. It was also noted that our population presented with more advanced disease as evidenced by clinical stage and PSA values.

## Figures and Tables

**Table 1:** Distribution of cancers by tumor differentiation.

Gleason score (level of differentiation)	Indo-Trinidadian number (%)	Afro-Trinidadian number (%)	Other numbers (%)	Total	*p* value
≤6 (well)	19 (26.7)	23 (11.3)	1 (12.5)	43 (15.2)	0.01
7 (moderate)	34 (47.8)	91 (44.6)	4 (50)	129 (45.6)	—
8–10 (poor)	18 (26.3)	90 (44.1)	3 (37.5)	111 (39.2)	0.01

Total	71	204	8	283	

**Table 2:** Distribution of prostate cancer patients by D'Amico risk group, *n* = 247.

D'Amico risk classification	Indo-Trinidadian number (%)	Afro-Trinidadian number (%)	Other numbers (%)	Total	*p* value
High	32 (50.8)	121 (68.0)	3 (50.0)	156 (63.1)	0.04
Intermediate	22 (34.9)	48 (27.0)	3 (50.0)	73 (29.6)	—
Low	9 (14.3)	9 (5.0)	0 (0)	18 (7.3)	0.04

Total	63	178	6	247	

**Table 3:** Racial disparities in clinicopathologic features within the prostate.

	Indo-Trinidadian (%)	Afro-Trinidadian (%)	*p* value
PSA > 100 ng/mL	10.6	27.5	<0.001
D'Amico high risk	68.0	50.8	0.04
Poorly differentiated tumors	25.0	44.1	0.01
Perineural invasion	11.3	5.6	0.353
